# Development of new NGLY1 assay systems – toward developing an early screening method for NGLY1 deficiency

**DOI:** 10.1093/glycob/cwae067

**Published:** 2024-08-29

**Authors:** Hiroto Hirayama, Haruhiko Fujihira, Tadashi Suzuki

**Affiliations:** Glycometabolic Biochemistry Laboratory, RIKEN Cluster for Pioneering Research (CPR), Riken, 2-1 Hirosawa, Wako Saitama 351-0198, Japan; Glycometabolic Biochemistry Laboratory, RIKEN Cluster for Pioneering Research (CPR), Riken, 2-1 Hirosawa, Wako Saitama 351-0198, Japan; Glycometabolic Biochemistry Laboratory, RIKEN Cluster for Pioneering Research (CPR), Riken, 2-1 Hirosawa, Wako Saitama 351-0198, Japan

**Keywords:** ELISA, FRET, luciferase assay, NGLY1, Peptide: *N*-glycanase

## Abstract

Cytosolic peptide: *N*-glycanase (PNGase/NGLY1 in mammals) is an amidase (EC:3.5.1.52) widely conserved in eukaryotes. It catalyzes the removal of *N*-glycans on glycoproteins, converting *N*-glycosylated Asn into Asp residues. This enzyme also plays a role in the quality control system for nascent glycoproteins. Since the identification of a patient with an autosomal recessive genetic disorder caused by *NGLY1* gene dysfunction, known as NGLY1 deficiency or NGLY1 congenital disorder of deglycosylation (OMIM: 615273), in 2012, more than 100 cases have been reported worldwide. NGLY1 deficiency is characterized by a wide array of symptoms, such as global mental delay, intellectual disability, abnormal electroencephalography findings, seizure, movement disorder, hypolacrima or alacrima, and liver dysfunction. Unfortunately, no effective therapeutic treatments for this disease have been established. However, administration of adeno-associated virus 9 (AAV9) vector harboring human *NGLY1* gene to an NGLY1-deficient rat model (*Ngly1*^−/−^ rat) by intracerebroventricular injection was found to drastically improve motor function defects. This observation indicated that early therapeutic intervention could alleviate various symptoms originating from central nervous system dysfunction in this disease. Therefore, there is a keen interest in the development of facile diagnostic methods for NGLY1 deficiency. This review summarizes the history of assay development for PNGase/NGLY1 activity, as well as the recent progress in the development of novel plate-based assay systems for NGLY1, and also discusses future perspectives.

## Introduction

**Fig. 1:**
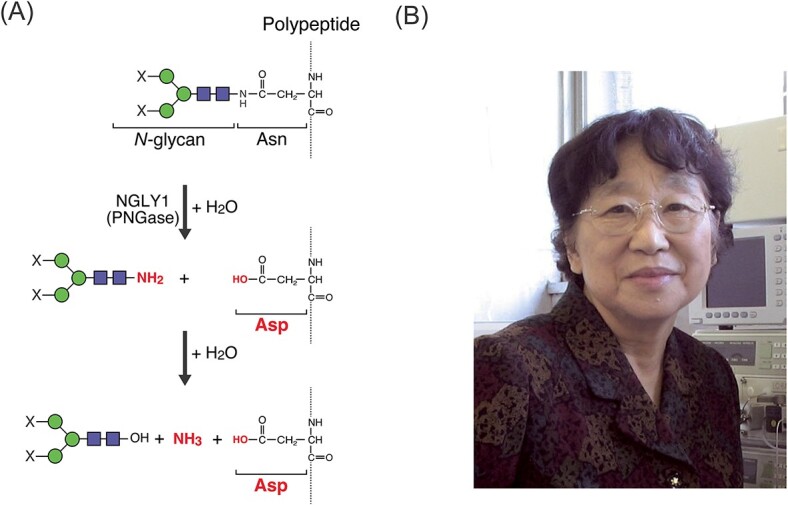
Enzyme reaction of peptide: * N*-glycanase. A) The reaction catalyzed by PNGase/NGLY1 can be divided into two steps. Molecules and groups generated in each reaction are shown in bold red text. X represents high mannose, hybrid, and complex-type *N*-glycans. Green circles and blue squares represent mannose and GlcNAc, respectively. B) Dr. Noriko Takahashi, a pioneering Japanese female scientist, first reported PNGase activity in almond emulsin.

Cytosolic peptide:*N-*glycanase (PNGase/NGLY1 in mammals) is an amidase (EC:3.5.1.52) that is widely conserved in eukaryotes. It catalyzes the removal of *N-*glycans on the consensus sequences (Asn-Xaa-Ser/Thr; Xaa is any amino acid except Pro) of glycoproteins, converting *N*-glycosylated Asn into Asp residues through deglycosylation (Fig. 1A). Moreover, this enzyme plays a role in the quality control system for nascent glycoproteins (Suzuki et al. 2002a). In particular, most nascent proteins in the endoplasmic reticulum (ER) are *N*-glycosylated by oligosaccharyltransferase-dependent glycan transfer from donor substrate (i.e. lipid-linked oligosaccharides in the ER membrane) (Harada Y. et al. 2015; Kornfeld and Kornfeld 1985; Lehle et al. 2006). These immature glycoproteins are then assisted by ER-resident molecular chaperones (such as Bip, calnexin/calreticulin, and protein disulfide isomerases) to achieve correct folding, allowing their transport to their appropriate destinations. Conversely, some fractions of nascent proteins that fail to achieve correct folding are recognized by the ER quality control system and are retrotranslocated to the cytosol. The misfolded glycoproteins thus delivered to the cytosol are deglycosylated by PNGase/NGLY1 when being degraded by the ubiquitin–proteasome system (Xu and Ng 2015; Ninagawa et al. 2021; Suzuki et al. 2022). It has been proposed that removing bulky *N*-glycans from misfolded proteins can facilitate their efficient degradation by the proteasome (Hirayama et al. 2015).

Since the identification of a patient with an autosomal recessive genetic disorder caused by *NGLY1* gene dysfunction, known as NGLY1 deficiency or NGLY1 congenital disorder of deglycosylation (NGLY1-CDDG) [OMIM: 615273], in the US in 2012 (Need et al. 2012), more than 100 cases have been reported worldwide, including in Europe, North and South America, Australia, India, China, and Japan (Pandey and Jafar-Nejad 2022; Sonoda et al. 2023). This disorder manifests a broad spectrum of symptoms, including global developmental delay and/or intellectual disability, abnormal electroencephalography (EEG) findings, seizures, movement disorders, hypolacrima or alacrima, and liver dysfunction (Need et al. 2012; Enns et al. 2014; Lam et al. 2017; van Keulen et al. 2019; Ge et al. 2020; Lipari Pinto et al. 2020; Kariminejad et al. 2021; Sonoda et al. 2023). Although some aspects of the molecular pathology of NGLY1-CDDG remain unclear, recent studies have disclosed the versatile functions of NGLY1 in various biological processes, which are well-summarized in recent review articles (Pandey et al. 2022; Suzuki and Fujihira 2024). These functions include not only facilitating the degradation of misfolded proteins but also activating the glycosylated transcription factor, nuclear factor erythroid 2-like 1 (NFE2L1), by editing its *N*-glycosylated Asn into Asp (Lehrbach and Ruvkun 2016; Tomlin et al. 2017; Lehrbach et al. 2019; Lehrbach 2022; Tachida et al. 2023), thereby aiding in the retrotranslocation of misfolded proteins from the ER to the cytosol (Galeone et al. 2020), maintaining mitochondrial homeostasis (Kong et al. 2018; Yang et al. 2018), innate immunity related to the cyclic GMP-AMP synthase (cGAS)–stimulator of interferon genes pathway (Yang et al. 2018), AMPK signaling pathway (Han et al. 2020), BMP signaling (Galeone et al. 2017; Galeone et al. 2020), preventing the accumulation of ubiquitinated *N*-glycoproteins (Yoshida et al. 2021), and supporting the maturation of neuronal cells during early brain development (Lin et al. 2022; Abbott et al. 2023).

Unfortunately, no effective therapeutic treatments for this disease have been established. Nevertheless, it is presumed that enzyme replacement and/or gene therapies could be effective for patients because (i) almost all *NGLY1* mutation alleles derived from patients lack enzyme activity (Hirayama and Suzuki 2022) and (ii) most symptoms appear to be associated with dysfunction of the central nervous system (CNS). Recent studies have demonstrated that administration of an adeno-associated viral vector serotype 9 (AAV9) harboring the human *NGLY1* gene to 3 or 5–7 week-old *NGLY1*-deficient model rats (*Ngly1*^−/−^ rats) by intracerebroventricular injection drastically improved motor function defects in the rats (Asahina et al. 2021; Fujihira et al. 2022; Zhu et al. 2022). On the basis of these findings, the FDA has granted investigational new drug clearance for the intracerebroventricular administration of AAV9 gene therapy, GS-100, to patients with NGLY1 deficiency (ClinicalTrials.gov ID: NCT06199531). Considering the effective therapeutic time windows for gene therapies, early therapeutic intervention could be critical to alleviate the various symptoms caused by CNS dysfunction in this disease. Therefore, there is a keen interest in the development of facile diagnostic methods for NGLY1 deficiency. Two different approaches are available for developing diagnostic methods for this disease, viz., (i) direct measurement of endogenous NGLY1 activity in patient specimens (e.g. fibroblasts and peripheral blood mononuclear cells (PBMCs)) and (ii) detection of potential NGLY1-specific biomarkers, such as Neu5Ac-Hex-GlcNAc-Asn (Hall et al. 2018) and GlcNAc-Asn (Haijes et al. 2019; Asahina et al. 2021; Mueller et al. 2022). In this regard, it is remarkable that novel facile NGLY1/PNGase assay methods, compatible with the 96-well format, have been reported using extracts from cells or tissues as an enzyme source (Takahashi et al. 2022; Fujihira et al. 2024; Hirayama et al. 2024). This review summarizes the history of assay development for PNGase/NGLY1, as well as newly developed assay systems, and discusses future perspectives.

## Early history of PNGase/NGLY1 assay system

Historically, Dr. Noriko Takahashi, a pioneering Japanese female scientist, first reported PNGase activity in almond emulsin (Takahashi 1977) (Fig. 1B). She conducted meticulous biochemical experiments to detect liberated glycans as well as deglycosylated peptides and found that almond PNGase catalyzed deglycosylation and the conversion of glycosylated Asn into Asp, thereby demonstrating that this enzyme is actually an amidase (i.e. cleaving an amide bond), not a glycosidase. As summarized in Table 1, various assays for PNGase activity have been established since the report of PNGase presence in nature. In the early era of PNGase research, it was essential to confirm two different reaction products to unequivocally define PNGase activity, viz., (i) liberated *N-*glycans from glycopeptide/protein substrates and (ii) peptides possessing Asp residues converted from *N*-glcosylated Asn, through laborious biochemical experiments. Various methods were applied for identifying the liberated *N*-glycans from the substrates, such as coloring reaction using the phenol/sulfuric acid method (Plummer et al. 1984; Taga et al. 1984; Chu 1986), quantification of 1-NH_2_ group in the liberated *N-*glycans (Takahashi and Nishibe 1981; Taga et al. 1984), composition analysis of released sugars (Plummer and Tarentino 1981; Taga et al. 1984; Tarentino et al. 1985; Chu 1986; Seko et al. 1991; Suzuki et al. 1993; Suzuki et al. 1994; Seko et al. 1999), and quantification of the glycans by labeling the reducing end sugar with NaB^3^H_4_ (Takahashi and Nishibe 1981; Hirani et al. 1987; Seko et al. 1991; Seko et al. 1999). More recently, a colorimetric detection method for the reducing ends of released glycans using a tetrazolium compound (WST1) was also developed (Wang et al. 2019). Furthermore, two major assay methods have been used for detecting deglycosylated peptides, viz., (i) detection and identification of the deglycosylated peptides labeled with radioisotopes (^3^H or ^14^C) (Takahashi 1977; Plummer and Tarentino 1981; Plummer et al. 1984; Tarentino et al. 1985; Chu 1986; Seko et al. 1991; Suzuki et al. 1993; Kitajima et al. 1995; Suzuki et al. 1997; Suzuki et al. 1998; Seko et al. 1999; Suzuki et al. 2000) or with fluorescence (Taga et al. 1984) at their N-termini and (ii) quantification of amino acid composition in the deglycosylated peptides using an amino acid analyzer (Plummer and Tarentino 1981; Takahashi and Nishibe 1981; Plummer et al. 1984; Taga et al. 1984; Chu 1986; Seko et al. 1991; Suzuki et al. 1993).

**Table 1:** Summary of assays for investigating PNGase/NGLY1 activity.

**Substrate for the assay**	**Detection method**	**Enzyme source used in the studies**	**References**
Glycopeptides from various glycoproteins (i.e. stem bromelain, ovalbumin, ovotransferrin)	Paper electrophoresis	Almond emulsin	(Takahashi 1977; Takahashi and Nishibe 1981)
Glycopeptide from ovalbumin and IgM	Amino acid analyzer	Almond emulsin	(Plummer and Tarentino 1981)
Bromelain glycopeptide, ovomucoid glycopeptide from turkey, dabsyl-labeled fetuin and ovalbumin peptide	Reversed-phase HPLC (detected by UV absorbance)	Almond emulsin, *Elizabethkingia meningoseptica*	(Plummer et al. 1984; Taga et al. 1984; Tretter et al. 1991)
Fetuin, transferrin, invertase, ribonuclease B (RNaseB), α-1-acid glycoprotein	SDS-PAGE and lectin blot	*E. meningoseptica*	(Tarentino et al. 1985; Chu 1986; Hirani et al. 1987; Suzuki 2005; Tanabe et al. 2006; Hosomi et al. 2010; Huang et al. 2015)
Ovomucoid glycopeptide from turkey	^1^H NMR	Almond emulsin	(Risley and Van Etten 1985)
Fetuin asialoglycopeptide and hyosophorin	Paper electrophoresis/paper chromatography	Medaka fish, mouse tissue, and mammalian cultured cells	(Seko et al. 1991; Suzuki et al. 1993; Suzuki et al. 1994; Kitajima et al. 1995; Seko et al. 1999)
Nontoxic mutant of ricin A subunit (RTA∆)^a^	SDS-PAGE and immunoblot	Mammalian cells and yeast	(Tanabe et al. 2006; Hosomi et al. 2010; Masahara-Negishi et al. 2012; Huang et al. 2015; Galeone et al. 2017)
ddVenus	FACS or microscopies	Culture cell lines	(Grotzke et al. 2013)
Horseradish peroxidase, RNaseB	Colorimetric assay	Recombinant PNGase F (expressed in *E. coli*)	(Wang et al. 2019)
5FAM-glycosylated cyclopeptide (5FAM-GCP)	HPLC	Cell-free system, fibroblast, blood cells, and culture cell lines	(Hirayama et al. 2022)
GlcNAc_2_-modified split intein fused with NanoLuc	NanoLuc luciferase	Purified *Sc*Png1, culture cell lines	(Takahashi et al. 2022)
FRET-based GCP probe (fGCP)	FRET	Fibroblast, blood cells, culture cell lines, and rat tissues	(Hirayama et al. 2024)
BSA glycopeptide-tag substrate (BSA-Gp)	ELISA	Human fibroblast and culture cells	(Fujihira et al. 2024)

a It should be noted that the occurrence of “nonglycosylated form” does not necessarily confirm the deglycosylation by NGLY1 (Huang and Suzuki 2020).

After demonstrating the enzymes as PNGases, two methods were primarily applied to routinely detect PNGase activity. One method involves assays using ^14^C-labeled glycopeptide prepared from bovine fetuin (^14^C-labeled asialofetuin) as a substrate. In this system, the substrate was reacted with enzyme sources, separated by paper chromatography or paper electrophoresis, and the reaction product was detected by autoradiography (Hirani et al. 1987; Seko et al. 1991; Suzuki et al. 1993; Suzuki et al. 1994; Kitajima et al. 1995; Suzuki et al. 1997; Suzuki et al. 1998; Seko et al. 1999; Suzuki et al. 2000). One advantage of using fetuin with crude extracts of animal origin as an enzyme source is that its glycan structure (tri (2/4, 2)-antennary complex-type glycans) is resistant to the action of endo-β-*N*-acetylglucosaminidase (ENGase) (Tachibana et al. 1982), another cytosolic deglycosylating enzyme that acts on *N*-glycans (Suzuki et al. 2002b). This method enables PNGase researchers to identify PNGase activities in various organisms. The other method uses *S*-alkylated RNaseB (alk-RNaseB), which has its disulfide bonds reduced and thiol groups alkylated by iodoacetamide, as a substrate (Tarentino et al. 1985; Chu 1986; Hirani et al. 1987; Hirsch et al. 2004; Suzuki 2005). In this assay, after the incubation of alk-RNaseB with the enzyme source, the substrate and product are separated by SDS-PAGE and detected by CBB staining or immunoblotting. Although this assay is semiquantitative, it remains popular for measuring PNGase activity because the product can be easily detected using commonly used techniques (i.e. SDS-PAGE followed by CBB staining or immunoblotting).

To detect intracellular NGLY1 activity in living cells, a reporter fluorescent protein known as deglycosylation-dependent Venus (ddVenus) was also reported (Grotzke et al. 2013). ddVenus consists of an ER-entry signal sequence and a mutant Venus protein, in which the DFT sequence at positions 82–84 is substituted with an *N-*glycosylation site (NFT). This protein becomes a functional fluorescent protein only when it is initially glycosylated in the ER and subsequently undergoes NGLY1-dependent editing of its *N*-glycosylated Asn into Asp (N^82^FT to D^82^FT). To evaluate the intracellular activity of NGLY1 in patient-derived fibroblasts, these cells were transfected with ddVenus-expressing plasmids. After transfection, the fibroblasts were treated with the proteasome inhibitor MG132 to enhance the fluorescent signal of deglycosylated ddVenus. However, FACS-based population analysis by measuring the fluorescence intensity of ddVenus was required to evaluate NGLY1 activity because of the variable level of fluorescence among cells (Grotzke et al. 2013; He et al. 2015).

**Fig. 2:**
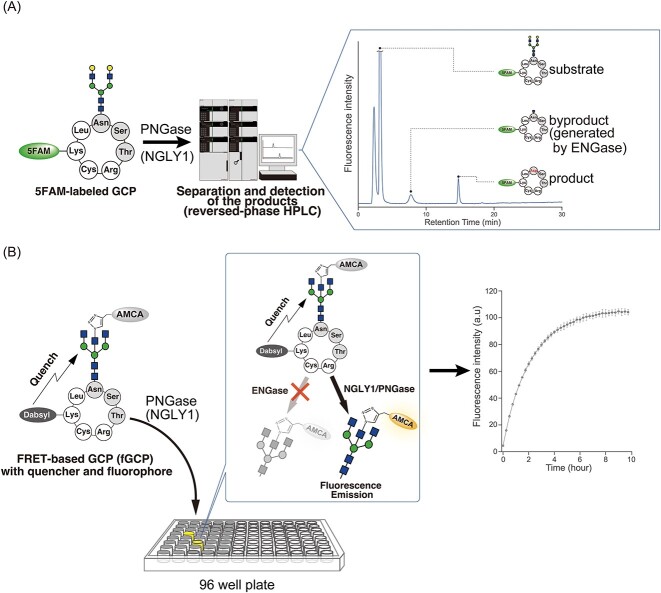
Development of 5FAM-GCP assay to FRET-based GCP assay (Hirayama et al. 2022; Hirayama et al. 2024). A) Schematic of assaying NGLY1 activity using 5FAM-GCP. 5FAM-GCP and the reaction were separated and quantified by HPLC. B) Detection of NGLY1 activity using a FRET-based probe. NGLY1 activity was detected by real-time measurement of fluorescence using a plate reader. Green circle and blue square represent mannose and GlcNAc, respectively.

Since the first report of the patient with NGLY1 deficiency in 2012, developing quantitative assay methods for measuring endogenous NGLY1 activity in cells/tissues extracts has become a critical need for NGLY1 research. Unfortunately, the previously used methods, such as those involving ^14^C-labeled asialofetuin peptide or alk-RNaseB, are not suitable for diagnosing this disease. For instance, the preparation of ^14^C-labeled asialofetuin and the use of radioactive molecules make it challenging to perform this assay in a standard laboratory. Moreover, alk-RNaseB can be susceptible to proteolytic degradation by endogenous peptidases/proteases present in crude enzyme sources (e.g. cell or tissue extracts), thus compromising the precise detection of endogenous PNGase/NGLY1 activity. Therefore, it was imperative to establish quantitative methods applicable for diagnosis. Recently, our research group investigated various chemically synthesized *N*-glycopeptides and found that 5FAM-labeled glycosylated hepta-cyclopeptides (5FAM-GCP) can be used to measure endogenous NGLY1 activity in crude extracts without the generation of subproducts (i.e. proteolytically degraded deglycosylated peptides by the contaminating protease/peptidase) (Hirayama et al. 2022; Hirayama and Suzuki 2022) (Fig. 2A). This was accomplished by separating the substrate and product by HPLC to confirm that essentially no proteolytic degradation product was detected. Furthermore, endogenous NGLY1 activity in fibroblasts and PBMCs could be quantified using 5FAM-GCP, indicating that the assay using 5FAM-GCP could be a useful tool for evaluating NGLY1 activity in patient-derived cells. Nonetheless, this assay requires product separation and quantitation by HPLC, which is often unavailable in standard clinical laboratories. Therefore, it was further required to develop an HPLC-free NGLY1 assay system.

## Development of novel microplate-based quantitative assay systems for measuring endogenous NGLY1 activity in cells and tissues

Recent studies have reported three novel, microplate-compatible NGLY1 assays (i.e. FRET-based GCP assay, PTS-based luciferase assay, and ELISA-based BSA-Gp assay) (Table 2) (Takahashi et al. 2022; Fujihira et al. 2024; Hirayama et al. 2024). The details of these three assays will be summarized in the following sections.

**Table 2:** Comparison table for the three different plate-based methods for measuring endogenous NGLY1 activity.

	**FRET-based assay**	**PTS-based split Luc assay**	**ELISA-based assay**
Probe used in each assay	fGCP	NLs-3GN2-IntN and IntCmut3-NLlg	BSA-Gp
Availability of the probe	Commercially available (GlyTech. Inc)	*Not for sale*	Commercially available (GlyTech. Inc)
Number of cells required for one reaction	*1–5 × 10^6^ cells*	8 × 10^3^ cell/well (HeLa) 1.5 × 10^4^ cells/well (3T3-L1)	**5 × 10^3^ cells**
Reaction time (h)	0.5–12	0.5–8	1.0–16
Applied for live imaging	Not applicable	**Possible**	Not applicable
Detection	Fluorescence	Luciferase activity (luminescence)	Anti-HA, and anti-IgG conjugated with HRP
Background signal	Weak	Weak	*Modest*
Resistance against ENGase	**Excellent**	**Excellent**	Not determined
Resistance against proteases	**Excellent**	Potentially problematic	Potentially problematic
Reference	(Hirayama et al. 2024)	(Takahashi et al. 2022)	(Fujihira et al. 2024)

(Pros and cons of each assay are denoted by *bold letters with underlining* and *italic letters with underlining*, respectively.)

### FRET-based GCP assay for measuring NGLY1 activity

Encouraged by various FRET-based fluorescence-quenching systems, such as a FRET probe for detecting ENGase activity (MANT-M3GN2-DNP) (Ishii et al. 2023) and a FRET-based assay for protease activity (Bickett et al. 1993; Tanskul et al. 2003), a novel FRET-based assay for measuring endogenous NGLY1 activity has been developed by further improving the 5FAM-GCP assay (Hirayama et al. 2024). In the previous 5FAM-GCP assay, not only the NGLY1-dependent product (deglycosylated GCP) but also a byproduct, *N*-GlcNAc-GCP, generated by endogenous ENGase, was produced when the assay was performed using crude cell extracts (Fig. 2A). The generation of *N*-GlcNAc-GCP will cause a problem in the development of FRET-based probes because the ENGase-dependent cleavage of *N-*glycans might also produce FRET-based fluorescence, thereby causing high nonspecific signals. To overcome this problem, the researchers exploited the fact that ENGase generally has narrower substrate specificities than NGLY1/PNGase (Tachibana et al. 1982; Maley et al. 1989; Suzuki et al. 1994; Tarentino and Plummer Jr 1994; Suzuki et al. 1995; Murakami et al. 2013; Hirayama et al. 2024). In the recent study, the researchers constructed a FRET-based GCP probe (fGCP) that exhibited strong resistance to the action of ENGase (Hirayama et al. 2024). fGCP consists of a glyco-cyclopeptide, including a lysine residue derivatized with a quencher, dabcyl, and an agalacto-biantenna glycan labeled with a fluorophore, AMCA, via bisected GlcNAz (Fig. 2B). Although a relatively larger amount of cells was required for this assay (1–5 × 10^6^ cells), it enabled microplate-based, real-time measurement of endogenous NGLY1 activity in various enzyme sources, including cell lines, rodent tissues, and PBMCs (Hirayama et al. 2024). The researchers also demonstrated that the fGCP assay could quantitatively measure NGLY1 activity in NGLY1-deficient, patient-derived fibroblasts, in which NGLY1 activity was severely compromised (Hirayama et al. 2024). These findings indicate that fGCP is a powerful tool for characterizing endogenous NGLY1 activities in patient-derived cells.

### Protein trans-splicing-based luciferase assay for measuring NGLY1 activity

**Fig. 3:**
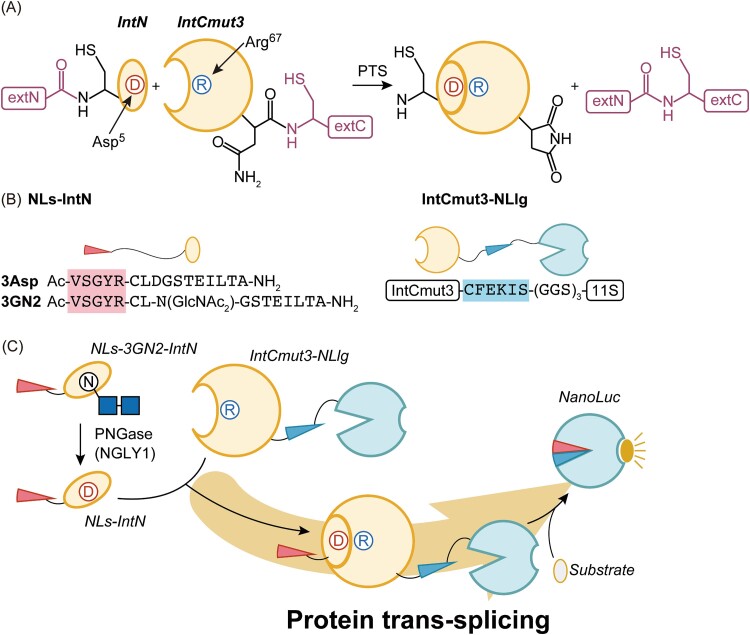
Measurement of NGLY1 activity by PTS-based luciferase assay (Takahashi et al. 2022). A) Schematic of PTS using engineered split inteins. Asp^5^ in IntN and Arg^67^ in IntCmut3 enhance their interaction. B) Amino acid sequences and the structure of NLS-IntN and IntCmut3-NLlg. 11S represents codon-optimized N-terminus fragment of NanoLuc 1-156 (Dixon et al. 2016). Sequences shaded by red and blue represent the C-terminal short peptide of NanoLuc, which can interact with 11S. C) PTS-based luciferase assay for detecting PNGase/NGLY1 activity. Blue square represents GlcNAc.

In a separate study, (Takahashi et al. 2022) demonstrated a novel protein trans-splicing (PTS)-based luciferase assay for measuring NGLY1 activity (Takahashi et al. 2022). They observed that intein is responsible for self-catalytic PTS, wherein an internal protein segment (intein) can excise itself and connect the remaining proteins, the exteins, with a peptide bond (Skretas and Wood 2005; Sakamoto et al. 2013; Takahashi and Saito 2016; Gramespacher et al. 2017). The authors previously demonstrated that PTS is also mediated by a pair of split inteins, IntN and IntCmut3, derived from *Nostoc punctiforme* (*Npu*) DnaE (Kawase et al. 2021) (Fig. 3A). They also showed that the interaction of a pair of fusion proteins, the C-terminal region of NanoLuc fused with IntN (NLs-IntN) and IntCmut3 fused with a NanoLuc fragment, 11S (IntCm3-NLlg) (Fig. 3B), promoted PTS and generated active NanoLuc (Fig. 3C) (Takahashi et al. 2022).

**Fig. 4:**
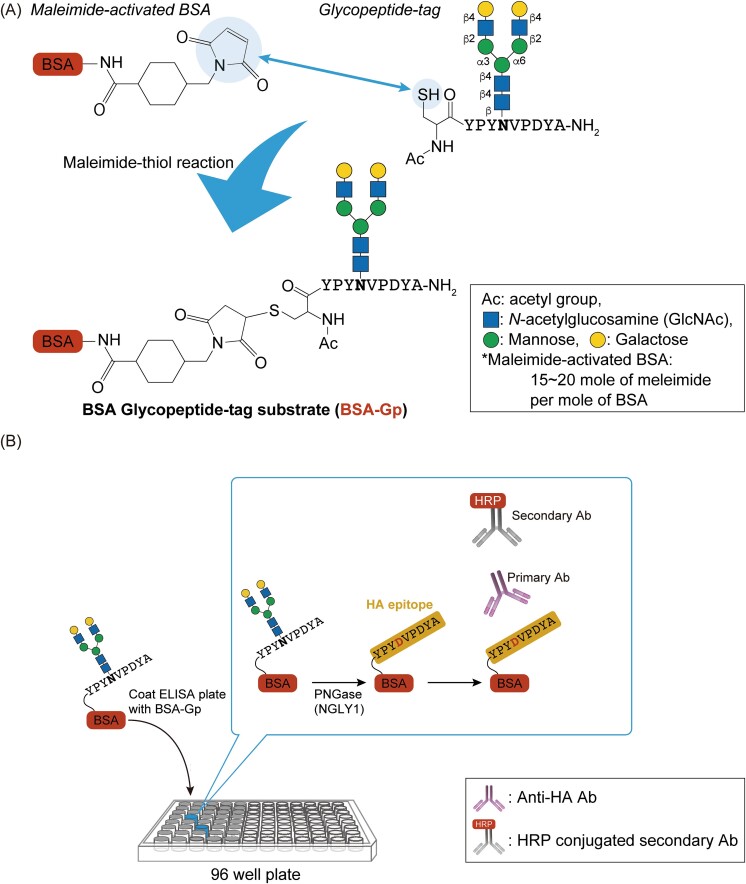
ELISA-based NGLY1 assay system (Fujihira et al. 2024). A) Schematic of the synthesis of BSA-Gp by maleimide-thiol reaction. N- and C-termini of glycopeptide-tag were modified with acetyl and amide-group, respectively, to confer the peptide some resistance against proteases. B) Schematic of ELISA-based NGLY1 assay system. The conversion of amino acid where the *N-*glycosylated Asn to asp turns into HA epitope. HRP represents horseradish peroxidase.

To develop their split intein system for a read-out of NGLY1 activity, the authors prepared NLs-3GN2-IntN, in which a GlcNAc_2_ moiety is incorporated into NLs-IntN. In this system, active NanoLuc is generated in a PTS-dependent manner only when Asn^67^-GlcNAc_2_, which is the minimum glycan structure serving as a PNGase/NGLY1 substrate, is converted into Asp^67^ by the action of PNGase/NGLY1. Using this setup, they successfully detected PNGase/NGLY1 activity with luciferase-derived luminescence as a read-out, by incubating enzyme sources (e.g. purified yeast Png1 and cytosolic fractions from cultured cell lines) with chemically synthesized NLs-3GN2-IntN and recombinantly expressed IntCm3-NLlg proteins (Takahashi et al. 2022) (Fig. 3C). The authors further developed this system for monitoring NGLY1 activity in living cells. In this system, they prepared NLs-3GN2-R9, which incorporates a stretch of nine arginine residues into GN2-IntN to facilitate its endocytotic transport into cells of interest. By treating cells that exogenously express IntCm3-NLlg with the NLs-3GN2-R9 peptide and a substrate for luciferase, furimazine, the authors were able to detect intracellular NGLY1 activity in cultured cell lines, HeLa and 3T3-L1 cells (Takahashi et al. 2022). These data indicate the usefulness of their assay system for detecting endogenous NGLY1 activity both *in vitro* and *in cellulo*.

### ELISA-based BSA-Gp assay for measuring NGLY1 activity

A recent study reported a novel ELISA-based NGLY1 assay through the detection of NGLY1-dependent amino acid editing (i.e. conversion of *N*-glycosylated Asn into Asp) (Fujihira et al. 2024). In that study, a glycopeptide-tag, in which the Asp residue at the 4th position in the hemagglutinin tag (HA-tag; YPY**D**VPDYA) was substituted with *N*-glycosylated Asn (YPY**N (glycans)**VPDYA), was chemically synthesized (Fig. 4A). The glycopeptides were conjugated, through the thiol group at the N-terminus cysteine, with a carrier protein, maleimide-activated BSA, via a maleimide-thiol reaction to synthesize the BSA glycopeptide-tag substrate (BSA-Gp) (Fig. 4A). After confirming the detection of the HA-epitope in BSA-Gp treated with PNGase/NGLY1 by immunoblotting, the authors demonstrated that BSA-Gp coated onto a 96-well plate can specifically detect PNGase/NGLY1 activity through the ELISA-based system only when the HA epitope is exposed through PNGase/NGLY1-specific editing of *N*-glycosylated Asn into Asp (Fig. 4B) (Fujihira et al. 2024). Remarkably, this assay system can detect endogenous NGLY1 activity in a highly sensitive manner, only requiring cell extracts from as little as 5 × 10^3^ cells (equivalent to 2 μg total protein). Moreover, the system is sensitive enough to detect weak endogenous NGLY1 activity in samples from patients with NGLY1 deficiency (~5% of the activity observed in healthy patients) (Fujihira et al. 2024). This observation strongly indicates that the ELISA-based assay system could be easily applied to clinical examinations for screening NGLY1 activity, as ELISAs are routinely applicable for clinical laboratory tests.

## Concluding remarks

This review summarizes the early history as well as recent advancements of the PNGase/NGLY1 assay system, particularly focusing on microplate-based assay systems for NGLY1 activity. The features and pros/cons of newly developed microplate-based assay systems are summarized in Table 2. Further refinement of the probes for real-time monitoring of intracellular NGLY1 activity in both cultured cell lines and tissues would provide powerful tools for gaining insights into the function and regulation of PNGase/NGLY1 activity across diverse organisms. Although each assay can quantitatively detect endogenous NGLY1 activity, it will be crucial to optimize the chemical structure of each probe for further improving sensitivity, a prerequisite for its diagnostic utility. In particular, further refinement of these assay systems to detect endogenous NGLY1 activity in a minute amount of blood-derived cells holds promise for establishing a highly effective diagnostic method for NGLY1 deficiency.

## References

[ref1] Abbott J, Tambe M, Pavlinov I, Farkhondeh A, Nguyen HN, Xu M, Pradhan M, York T, Might M, Baumgärtel K, et al. Generation and characterization of NGLY1 patient-derived midbrain organoids. Front Cell Dev Biol. 2023:11:1039182. 36875753 10.3389/fcell.2023.1039182 PMC9978932

[ref2] Asahina M, Fujinawa R, Hirayama H, Tozawa R, Kajii Y, Suzuki T. Reversibility of motor dysfunction in the rat model of NGLY1 deficiency. Mol Brain. 2021:14(1):91. 34120625 10.1186/s13041-021-00806-6 PMC8201687

[ref3] Bickett DM, Green MD, Berman J, Dezube M, Howe AS, Brown PJ, Roth JT, Mcgeehan GM. A high throughput fluorogenic substrate for interstitial collagenase (MMP-1) and gelatinase (MMP-9). Anal Biochem. 1993:212(1):58–64. 8368516 10.1006/abio.1993.1291

[ref4] Chu FK . Requirements of cleavage of high mannose oligosaccharides in glycoproteins by peptide *N*-glycosidase F. J Biol Chem. 1986:261(1):172–177. 3941069

[ref1d] Dixon AS, Schwinn MK, Hall MP, Zimmerman K, Otto P, Lubben TH, Butler BL, Binkowski BF, Machleidt T, Kirkland TA, et al. NanoLuc Complementation Reporter Optimized for Accurate Measurement of Protein Interactions in Cells. ACS Chem Biol, 2016:11(2):400–408. 10.1021/acschembio.5b00753 26569370

[ref5] Enns GM, Shashi V, Bainbridge M, Gambello MJ, Zahir FR, Bast T, Crimian R, Schoch K, Platt J, Cox R, et al. Mutations in NGLY1 cause an inherited disorder of the endoplasmic reticulum-associated degradation pathway. Genet Med. 2014:16(10):751–758. 24651605 10.1038/gim.2014.22 PMC4243708

[ref6] Fujihira H, Asahina M, Suzuki T. Physiological importance of NGLY1, as revealed by rodent model analyses. J Biochem. 2022:171(2):161–167. 34580715 10.1093/jb/mvab101

[ref7] Fujihira H, Sato K, Nishiuchi Y, Murase T, Matsuda Y, Yoshida Y, Kamei T, Suzuki T. ELISA-based highly sensitive assay system for the detection of endogenous NGLY1 activity. Biochem Biophys Res Commun. 2024:710:149826. 38581946 10.1016/j.bbrc.2024.149826

[ref8] Galeone A, Han SY, Huang C, Hosomi A, Suzuki T, Jafar-Nejad H. Tissue-specific regulation of BMP signaling by *drosophila N*-glycanase 1. elife. 2017:6:e27612. 28826503 10.7554/eLife.27612 PMC5599231

[ref9] Galeone A, Adams JM, Matsuda S, Presa MF, Pandey A, Han SY, Tachida Y, Hirayama H, Vaccari T, Suzuki T, et al. Regulation of BMP4/Dpp retrotranslocation and signaling by deglycosylation. elife. 2020:9:e55596. 32720893 10.7554/eLife.55596 PMC7394544

[ref10] Ge H, Wu Q, Lu H, Huang Y, Zhou T, Tan D, ZhongqinJin. Two novel compound heterozygous mutations in *NGLY1* as a cause of congenital disorder of deglycosylation: a case presentation. BMC Med Genet. 2020:21(1):135. 32576142 10.1186/s12881-020-01067-1 PMC7310492

[ref11] Gramespacher JA, Stevens AJ, Nguyen DP, Chin JW, Muir TW. Intein zymogens: conditional assembly and splicing of split Inteins via targeted proteolysis. J Am Chem Soc. 2017:139(24):8074–8077. 28562027 10.1021/jacs.7b02618 PMC5533455

[ref12] Grotzke JE, Lu Q, Cresswell P. Deglycosylation-dependent fluorescent proteins provide unique tools for the study of ER-associated degradation. Proc Natl Acad Sci USA. 2013:110(9):3393–3398. 23401531 10.1073/pnas.1300328110 PMC3587246

[ref13] Haijes HA, de M, Prinsen H, Willems AP, van M, Gerrits J, Couse MH, Friedman JM, van C, Selby KA, et al. Aspartylglycosamine is a biomarker for NGLY1-CDDG, a congenital disorder of deglycosylation. Mol Genet Metab. 2019:127(4):368–372. 31311714 10.1016/j.ymgme.2019.07.001

[ref14] Hall PL, Lam C, Alexander JJ, Asif G, Berry GT, Ferreira C, Freeze HH, Gahl WA, Nickander KK, Sharer JD, et al. Urine oligosaccharide screening by MALDI-TOF for the identification of NGLY1 deficiency. Mol Genet Metab. 2018:124(1):82–86. 29550355 10.1016/j.ymgme.2018.03.002 PMC10508399

[ref15] Han SY, Pandey A, Moore T, Galeone A, Duraine L, Cowan TM, Jafar-Nejad H. A conserved role for AMP-activated protein kinase in NGLY1 deficiency. PLoS Genet. 2020:16(12):e1009258. 33315951 10.1371/journal.pgen.1009258 PMC7769621

[ref16] Harada Y, Hirayama H, Suzuki T. Generation and degradation of free asparagine-linked glycans. Cell Mol Life Sci. 2015:72(13):2509–2533. 25772500 10.1007/s00018-015-1881-7 PMC11113800

[ref17] He P, Grotzke JE, Ng BG, Gunel M, Jafar-Nejad H, Cresswell P, Enns GM, Freeze HH. A congenital disorder of deglycosylation: biochemical characterization of *N*-glycanase 1 deficiency in patient fibroblasts. Glycobiology. 2015:25(8):836–844. 25900930 10.1093/glycob/cwv024 PMC4487302

[ref18] Hirani S, Bernasconi RJ, Rasmussen JR. Use of *N*-glycanase to release asparagine-linked oligosaccharides for structural analysis. Anal Biochem. 1987:162(2):485–492. 3605611 10.1016/0003-2697(87)90424-6

[ref19] Hirayama H, Suzuki T. Assay for the peptide:*N*-glycanase/NGLY1 and disease-specific biomarkers for diagnosing NGLY1 deficiency. J Biochem. 2022:171(2):169–176. 34791337 10.1093/jb/mvab127

[ref20] Hirayama H, Hosomi A, Suzuki T. Physiological and molecular functions of the cytosolic peptide:*N*-glycanase. Semin Cell Dev Biol. 2015:41:110–120. 25475175 10.1016/j.semcdb.2014.11.009

[ref21] Hirayama H, Tachida Y, Seino J, Suzuki T. A method for assaying peptide: *N*-glycanase/*N*-glycanase 1 activities in crude extracts using an *N*-glycosylated cyclopeptide. Glycobiology. 2022:32(2):110–122. 34939090 10.1093/glycob/cwab115 PMC8934141

[ref22] Hirayama H, Tachida Y, Fujinawa R, Matsuda Y, Murase T, Nishiuchi Y, Suzuki T. Development of a fluorescence and quencher-based FRET assay for detection of endogenous peptide:*N*-glycanase/NGLY1 activity. J Biol Chem. 2024:300(4):107121. 38417795 10.1016/j.jbc.2024.107121 PMC11065741

[ref23] Hirsch C, Misaghi S, Blom D, Pacold ME, Ploegh HL. Yeast *N*-glycanase distinguishes between native and non-native glycoproteins. EMBO Rep. 2004:5(2):201–206. 14726951 10.1038/sj.embor.7400066 PMC1298977

[ref24] Hosomi A, Tanabe K, Hirayama H, Kim I, Rao H, Suzuki T. Identification of an Htm1 (EDEM)-dependent, Mns1-independent endoplasmic reticulum-associated degradation (ERAD) pathway in *Saccharomyces cerevisiae*: application of a novel assay for glycoprotein ERAD. J Biol Chem. 2010:285(32):24324–24334. 20511219 10.1074/jbc.M109.095919 PMC2915668

[ref25] Huang C, Suzuki T. The occurrence of nonglycosylated forms of *N*-glycoprotein upon proteasome inhibition does not confirm cytosolic deglycosylation. FEBS Lett. 2020:594(9):1433–1442. 31951015 10.1002/1873-3468.13734

[ref26] Huang C, Harada Y, Hosomi A, Masahara-Negishi Y, Seino J, Fujihira H, Funakoshi Y, Suzuki T, Dohmae N, Suzuki T. Endo-beta-*N*-acetylglucosaminidase forms *N*-GlcNAc protein aggregates during ER-associated degradation in *Ngly1*-defective cells. Proc Natl Acad Sci USA. 2015:112(5):1398–1403. 25605922 10.1073/pnas.1414593112 PMC4321286

[ref27] Ishii N, Muto H, Nagata M, Sano K, Sato I, Iino K, Matsuzaki Y, Katoh T, Yamamoto K, Matsuo I. A fluorogenic probe for core-fucosylated glycan-preferred ENGase. Carbohydr Res. 2023:523:108724. 36435009 10.1016/j.carres.2022.108724

[ref28] Kariminejad A, Shakiba M, Shams M, Namiranian P, Eghbali M, Talebi S, Makvand M, Jaeken J, Najmabadi H, Hennekam RC. NGLY1 deficiency: novel variants and literature review. Eur J Med Genet. 2021:64(3):104146. 33497766 10.1016/j.ejmg.2021.104146

[ref29] Kawase M, Fujioka M, Takahashi T. Activation of protease and luciferase using engineered Nostoc punctiforme PCC73102 DnaE Intein with altered split position. Chembiochem. 2021:22(3):577–584. 32969142 10.1002/cbic.202000609

[ref30] van Keulen BJ, Rotteveel J, Finken MJJ. Unexplained death in patients with NGLY1 mutations may be explained by adrenal insufficiency. Physiol Rep. 2019:7(3):e13979. 30740912 10.14814/phy2.13979 PMC6369059

[ref31] Kitajima K, Suzuki T, Kouchi Z, Inoue S, Inoue Y. Identification and distribution of peptide:*N*-glycanase (PNGase) in mouse organs. Arch Biochem Biophys. 1995:319(2):393–401. 7786020 10.1006/abbi.1995.1309

[ref32] Kong J, Peng M, Ostrovsky J, Kwon YJ, Oretsky O, McCormick EM, He M, Argon Y, Falk MJ. Mitochondrial function requires NGLY1. Mitochondrion. 2018:38:6–16. 28750948 10.1016/j.mito.2017.07.008 PMC6038697

[ref33] Kornfeld R, Kornfeld S. Assembly of asparagine-linked oligosaccharides. Annu Rev Biochem. 1985:54(1):631–664. 3896128 10.1146/annurev.bi.54.070185.003215

[ref34] Lam C, Ferreira C, Krasnewich D, Toro C, Latham L, Zein WM, Lehky T, Brewer C, Baker EH, Thurm A, et al. Prospective phenotyping of NGLY1-CDDG, the first congenital disorder of deglycosylation. Genet Med. 2017:19(2):160–168. 27388694 10.1038/gim.2016.75 PMC7477955

[ref35] Lehle L, Strahl S, Tanner W. Protein glycosylation, conserved from yeast to man: a model organism helps elucidate congenital human diseases. Angew Chem Int Ed Engl. 2006:45(41):6802–6818. 17024709 10.1002/anie.200601645

[ref36] Lehrbach NJ . NGLY1: insights from *Caenorhabditis elegans*. J Biochem. 2022:171(2):145–152. 34697631 10.1093/jb/mvab112

[ref37] Lehrbach NJ, Ruvkun G. Proteasome dysfunction triggers activation of SKN-1A/Nrf1 by the aspartic protease DDI-1. elife. 2016:5:e17721. 27528192 10.7554/eLife.17721 PMC4987142

[ref38] Lehrbach NJ, Breen PC, Ruvkun G. Protein sequence editing of SKN-1A/Nrf1 by peptide:*N*-Glycanase controls proteasome gene expression. Cell. 2019:177(3):737–750.e15. 31002798 10.1016/j.cell.2019.03.035 PMC6574124

[ref39] Lin VJT, Hu J, Zolekar A, Salick MR, Mittal P, Bird JT, Hoffmann P, Kaykas A, Byrum SD, Wang YC. Deficiency of *N*-glycanase 1 perturbs neurogenesis and cerebral development modeled by human organoids. Cell Death Dis. 2022:13(3):262. 35322011 10.1038/s41419-022-04693-0 PMC8942998

[ref40] Lipari Pinto P, Machado C, Janeiro P, Dupont J, Quintas S, Sousa AB, Gaspar A. NGLY1 deficiency-a rare congenital disorder of deglycosylation. JIMD Rep. 2020:53(1):2–9. 32395402 10.1002/jmd2.12108 PMC7203651

[ref41] Maley F, Trimble RB, Tarentino AL, Plummer TH Jr. Characterization of glycoproteins and their associated oligosaccharides through the use of endoglycosidases. Anal Biochem. 1989:180(2):195–204. 2510544 10.1016/0003-2697(89)90115-2

[ref42] Masahara-Negishi Y, Hosomi A, Della Mea M, Serafini-Fracassini D, Suzuki T. A plant peptide: *N*-glycanase orthologue facilitates glycoprotein ER-associated degradation in yeast. Biochim Biophys Acta. 2012:1820(10):1457–1462. 22659524 10.1016/j.bbagen.2012.05.009

[ref43] Mueller WF, Zhu L, Tan B, Dwight S, Beahm B, Wilsey M, Wechsler T, Mak J, Cowan T, Pritchett J, et al. GlcNAc-Asn (GNA) is a biomarker for NGLY1 deficiency. J Biochem. 2022:171(2):177–186. 34697629 10.1093/jb/mvab111 PMC8863169

[ref44] Murakami S, Takaoka Y, Ashida H, Yamamoto K, Narimatsu H, Chiba Y. Identification and characterization of endo-beta-*N*-acetylglucosaminidase from methylotrophic yeast *Ogataea minuta*. Glycobiology. 2013:23(6):736–744. 23436287 10.1093/glycob/cwt012

[ref45] Need AC, Shashi V, Hitomi Y, Schoch K, Shianna KV, McDonald MT, Meisler MH, Goldstein DB. Clinical application of exome sequencing in undiagnosed genetic conditions. J Med Genet. 2012:49(6):353–361. 22581936 10.1136/jmedgenet-2012-100819 PMC3375064

[ref46] Ninagawa S, George G, Mori K. Mechanisms of productive folding and endoplasmic reticulum-associated degradation of glycoproteins and non-glycoproteins. Biochim Biophys Acta Gen Subj. 2021:1865(3):129812. 33316349 10.1016/j.bbagen.2020.129812

[ref47] Pandey A, Jafar-Nejad H. Tracing the NGLY1 footprints: insights from *drosophila*. J Biochem. 2022:171(2):153–160. 34270726 10.1093/jb/mvab084 PMC9005052

[ref48] Pandey A, Adams JM, Han SY, Jafar-Nejad H. NGLY1 deficiency, a congenital disorder of Deglycosylation: from disease gene function to pathophysiology. Cells. 2022:11(7):1155. 10.3390/cells11071155 PMC8997433 35406718

[ref49] Plummer TH, Tarentino AL. Facile cleavage of complex oligosaccharides from glycopeptides by almond emulsin peptide: *N*-glycosidase. J Biol Chem. 1981:256(20):10243–10246. 7287707

[ref50] Plummer TH, Elder JH, Alexander S, Phelan AW, Tarentino AL. Demonstration of peptide:*N*-glycosidase F activity in endo-beta-*N*-acetylglucosaminidase F preparations. J Biol Chem. 1984:259(17):10700–10704. 6206060

[ref51] Risley JM, Van Etten RL. 1H NMR evidence that almond “peptide: *N*-glycosidase” is an amidase. Kinetic data and trapping of the intermediate. J Biol Chem. 1985:260(29):15488–15494. 4066679

[ref52] Sakamoto S, Terauchi M, Hugo A, Kim T, Araki Y, Wada T. Creation of a caspase-3 sensing system using a combination of split-GFP and split-intein. Chem Commun (Camb). 2013:49(87):10323–10325. 24067843 10.1039/c3cc43389g

[ref53] Seko A, Kitajima K, Inoue Y, Inoue S. Peptide:*N*-glycosidase activity found in the early embryos of *Oryzias latipes* (Medaka fish). The first demonstration of the occurrence of peptide:*N*-glycosidase in animal cells and its implication for the presence of a de-*N*-glycosylation system in living or. J Biol Chem. 1991:266(33):22110–22114. 1718990

[ref54] Seko A, Kitajima K, Iwamatsu T, Inoue Y, Inoue S. Identification of two discrete peptide: *N*-glycanases in *Oryzias latipes* during embryogenesis. Glycobiology. 1999:9(9):887–895. 10460830 10.1093/glycob/9.9.887

[ref55] Skretas G, Wood DW. Regulation of protein activity with small-molecule-controlled inteins. Protein Sci. 2005:14(2):523–532. 15632292 10.1110/ps.04996905 PMC2386410

[ref56] Sonoda Y, Fujita A, Torio M, Mukaino T, Sakata A, Matsukura M, Yonemoto K, Hatae K, Ichimiya Y, Chong PF, et al. Progressive myoclonic epilepsy as an expanding phenotype of NGLY1-associated congenital deglycosylation disorder: a case report and review of the literature. Eur J Med Genet. 2023:67:104895. 38070824 10.1016/j.ejmg.2023.104895

[ref57] Suzuki T . A simple, sensitive *in vitro* assay for cytoplasmic deglycosylation by peptide: *N*-glycanase. Methods. 2005:35(4):360–365. 15804608 10.1016/j.ymeth.2004.10.008

[ref58] Suzuki T, Fujihira H. NGLY1: a fascinating, multifunctional molecule. Biochim Biophys Acta Gen Subj. 2024:1868(2):130379. 37951368 10.1016/j.bbagen.2023.130379

[ref59] Suzuki T, Seko A, Kitajima K, Inoue Y, Inoue S. Identification of peptide:*N*-glycanase activity in mammalian-derived cultured cells. Biochem Biophys Res Commun. 1993:194(3):1124–1130. 8352768 10.1006/bbrc.1993.1938

[ref60] Suzuki T, Seko A, Kitajima K, Inoue Y, Inoue S. Purification and enzymatic properties of peptide:*N*-glycanase from C3H mouse-derived L-929 fibroblast cells. Possible widespread occurrence of post-translational remodification of proteins by *N*-deglycosylation. J Biol Chem. 1994:269(26):17611–17618. 8021270

[ref61] Suzuki T, Kitajima K, Inoue S, Inoue Y. *N*-glycosylation/deglycosylation as a mechanism for the post-translational modification/remodification of proteins. Glycoconj J. 1995:12(3):183–193. 7496130 10.1007/BF00731318

[ref62] Suzuki T, Kitajima K, Emori Y, Inoue Y, Inoue S. Site-specific de-*N*-glycosylation of diglycosylated ovalbumin in hen oviduct by endogenous peptide: *N*-glycanase as a quality control system for newly synthesized proteinsw. Proc Natl Acad Sci USA. 1997:94(12):6244–6249. 9177202 10.1073/pnas.94.12.6244 PMC21034

[ref63] Suzuki T, Park H, Kitajima K, Lennarz WJ. Peptides glycosylated in the endoplasmic reticulum of yeast are subsequently deglycosylated by a soluble peptide: *N*-glycanase activity. J Biol Chem. 1998:273(34):21526–21530. 9705282 10.1074/jbc.273.34.21526

[ref64] Suzuki T, Park H, Hollingsworth NM, Sternglanz R, Lennarz WJ. *PNG*1, a yeast gene encoding a highly conserved peptide:*N*-glycanase. J Cell Biol. 2000:149(5):1039–1052. 10831608 10.1083/jcb.149.5.1039 PMC2174826

[ref65] Suzuki T, Park H, Lennarz WJ. Cytoplasmic peptide:*N*-glycanase (PNGase) in eukaryotic cells: occurrence, primary structure, and potential functions. FASEB J. 2002a:16(7):635–641. 11978727 10.1096/fj.01-0889rev

[ref66] Suzuki T, Yano K, Sugimoto S, Kitajima K, Lennarz WJ, Inoue S, Inoue Y, Emori Y. Endo-beta-*N*-acetylglucosaminidase, an enzyme involved in processing of free oligosaccharides in the cytosol. Proc Natl Acad Sci USA. 2002b:99(15):9691–9696. 12114544 10.1073/pnas.152333599 PMC124980

[ref67] Suzuki T, Cummings RD, Aebi M, Parodi A. Glycans in glycoprotein quality control. In: Varki A, Cummings RD, Esko JD, Stanley P, Hart GW, Aebi M, Mohnen D, Kinoshita T, Packer NH, Prestegard JH, et al., editors. Essentials of Glycobiology. Cold Spring Harbor (NY): Cold Spring Harbor Laboratory; 2022. pp. 529–538 35536972

[ref68] Tachibana Y, Yamashita K, Kobata A. Substrate specificity of mammalian endo-beta-*N*-acetylglucosaminidase: study with the enzyme of rat liver. Arch Biochem Biophys. 1982:214(1):199–210. 6805439 10.1016/0003-9861(82)90023-6

[ref69] Tachida Y, Hirayama H, Suzuki T. Amino acid editing of NFE2L1 by PNGase causes abnormal mobility on SDS-PAGE. Biochim Biophys Acta Gen Subj. 2023:1867(12):130494. 37865174 10.1016/j.bbagen.2023.130494

[ref70] Taga EM, Waheed A, Van Etten RL. Structural and chemical characterization of a homogeneous peptide *N*-glycosidase from almond. Biochemistry. 1984:23(5):815–822. 6712926 10.1021/bi00300a006

[ref71] Takahashi N . Demonstration of a new amidase acting on glycopeptides. Biochem Biophys Res Commun. 1977:76(4):1194–1201. 901470 10.1016/0006-291x(77)90982-2

[ref72] Takahashi N, Nishibe H. Almond glycopeptidase acting on aspartylglycosylamine linkages. Multiplicity and substrate specificity. Biochim Biophys Acta. 1981:657(2):457–467. 7213757 10.1016/0005-2744(81)90331-4

[ref73] Takahashi T, Saito A. Interaction-dependent native chemical ligation and protein trans-splicing (IDNCL-PTS) for detection and visualization of ligand-protein interactions. Chemistryselect. 2016:1(8):1768–1772.

[ref74] Takahashi T, Uchibayashi T, Ishii N, Matsuo I, Yoshida Y, Suzuki T. Luminescence detection of peptide:*N*-glycanase activity using engineered split inteins. Chem Commun (Camb). 2022:58(95):13282–13285. 36373598 10.1039/d2cc04865e

[ref75] Tanabe K, Lennarz WJ, Suzuki T. A cytoplasmic peptide: *N*-glycanase. Methods Enzymol. 2006:415:46–55. 17116467 10.1016/S0076-6879(06)15004-1

[ref76] Tanskul S, Oda K, Oyama H, Noparatnaraporn N, Tsunemi M, Takada K. Substrate specificity of alkaline serine proteinase isolated from photosynthetic bacterium, Rubrivivax gelatinosus KDDS1. Biochem Biophys Res Commun. 2003:309(3):547–551. 12963024 10.1016/j.bbrc.2003.08.035

[ref77] Tarentino AL, Plummer TH Jr. Enzymatic deglycosylation of asparagine-linked glycans: purification, properties, and specificity of oligosaccharide-cleaving enzymes from *Flavobacterium meningosepticum*. Methods Enzymol. 1994:230:44–57. 8139511 10.1016/0076-6879(94)30006-2

[ref78] Tarentino AL, Gomez CM, Plummer TH. Deglycosylation of asparagine-linked glycans by peptide:* N*-glycosidase F. Biochemistry. 1985:24(17):4665–4671. 4063349 10.1021/bi00338a028

[ref79] Tomlin FM, Gerling-Driessen UIM, Liu YC, Flynn RA, Vangala JR, Lentz CS, Clauder-Muenster S, Jakob P, Mueller WF, Ordonez-Rueda D, et al. Inhibition of NGLY1 inactivates the transcription factor Nrf1 and potentiates proteasome inhibitor cytotoxicity. ACS Cent Sci. 2017:3(11):1143–1155. 29202016 10.1021/acscentsci.7b00224 PMC5704294

[ref80] Tretter V, Altmann F, Marz L. Peptide-*N*4-(*N*-acetyl-beta-glucosaminyl) asparagine amidase F cannot release glycans with fucose attached alpha 1→3 to the asparagine-linked *N*-acetylglucosamine residue. Eur J Biochem. 1991:199(3):647–652. 1868849 10.1111/j.1432-1033.1991.tb16166.x

[ref81] Wang T, Zheng SL, Liu L, Voglmeir J. Development of a colorimetric PNGase activity assay. Carbohydr Res. 2019:472:58–64. 30476755 10.1016/j.carres.2018.11.007

[ref82] Xu C, Ng DTW. Glycosylation-directed quality control of protein folding. Nat Rev Mol Cell Biol. 2015:16(12):742–752. 26465718 10.1038/nrm4073

[ref83] Yang K, Huang R, Fujihira H, Suzuki T, Yan N. *N*-glycanase NGLY1 regulates mitochondrial homeostasis and inflammation through NRF1. J Exp Med. 2018:215(10):2600–2616. 30135079 10.1084/jem.20180783 PMC6170171

[ref84] Yoshida Y, Asahina M, Murakami A, Kawawaki J, Yoshida M, Fujinawa R, Iwai K, Tozawa R, Matsuda N, Tanaka K, et al. Loss of peptide:N-glycanase causes proteasome dysfunction mediated by a sugar-recognizing ubiquitin ligase. Proc Natl Acad Sci USA. 2021:118(27):e2102902118. 34215698 10.1073/pnas.2102902118 PMC8271764

[ref85] Zhu L, Tan B, Dwight SS, Beahm B, Wilsey M, Crawford BE, Schweighardt B, Cook JW, Wechsler T, Mueller WF. AAV9-NGLY1 gene replacement therapy improves phenotypic and biomarker endpoints in a rat model of NGLY1 deficiency. Mol Ther Methods Clin Dev. 2022:27:259–271. 36320418 10.1016/j.omtm.2022.09.015 PMC9593239

